# scPlantLLM: A Foundation Model for Exploring Single-cell Expression Atlases in Plants

**DOI:** 10.1093/gpbjnl/qzaf024

**Published:** 2025-03-17

**Authors:** Guangshuo Cao, Haoyu Chao, Wenqi Zheng, Yangming Lan, Kaiyan Lu, Yueyi Wang, Ming Chen, He Zhang, Dijun Chen

**Affiliations:** State Key Laboratory of Pharmaceutical Biotechnology, School of Life Sciences, Nanjing University, Nanjing 210023, China; State Key Laboratory of Pharmaceutical Biotechnology, School of Life Sciences, Nanjing University, Nanjing 210023, China; Department of Bioinformatics, College of Life Sciences, Zhejiang University, Hangzhou 310058, China; Kuang Yaming Honors School, Nanjing University, Nanjing 210023, China; State Key Laboratory of Pharmaceutical Biotechnology, School of Life Sciences, Nanjing University, Nanjing 210023, China; State Key Laboratory of Pharmaceutical Biotechnology, School of Life Sciences, Nanjing University, Nanjing 210023, China; State Key Laboratory of Pharmaceutical Biotechnology, School of Life Sciences, Nanjing University, Nanjing 210023, China; Department of Bioinformatics, College of Life Sciences, Zhejiang University, Hangzhou 310058, China; Zhejiang Provincial Key Lab for Subtropical Water Environment and Marine Biological Resources Protection, College of Life and Environmental Science, Wenzhou University, Wenzhou 325035, China; State Key Laboratory of Pharmaceutical Biotechnology, School of Life Sciences, Nanjing University, Nanjing 210023, China; Chemistry and Biomedicine Innovation Center, Nanjing University, Nanjing 210023, China

**Keywords:** Single-cell RNA sequencing, Artificial intelligence, Foundation model, Gene regulatory network, Plant

## Abstract

Single-cell RNA sequencing (scRNA-seq) provides unprecedented insights into plant cellular diversity by enabling high-resolution analyses of gene expression at the single-cell level. However, the complexity of scRNA-seq data, including challenges in batch integration, cell type annotation, and gene regulatory network (GRN) inference, demands advanced computational approaches. To address these challenges, we developed scPlantLLM, a Transformer model trained on millions of plant single-cell data points. Using a sequential pretraining strategy incorporating masked language modeling and cell type annotation tasks, scPlantLLM generates robust and interpretable single-cell data embeddings. When applied to *Arabidopsis thaliana* datasets, scPlantLLM excels in clustering, cell type annotation, and batch integration, achieving an accuracy of up to 0.91 in zero-shot learning scenarios. Furthermore, the model demonstrates an ability to identify biologically meaningful GRNs and subtle cellular subtypes, showcasing its potential to advance plant biology research. Compared to traditional methods, scPlantLLM outperforms in key metrics such as adjusted rand index (ARI), normalized mutual information (NMI), and silhouette score (SIL), highlighting its superior clustering accuracy and biological relevance. scPlantLLM represents a foundation model for exploring plant single-cell expression atlases, offering unprecedented capabilities to resolve cellular heterogeneity and regulatory dynamics across diverse plant systems. The code used in this study is available at https://github.com/compbioNJU/scPlantLLM.

## Graphical abstract

**Figure:**
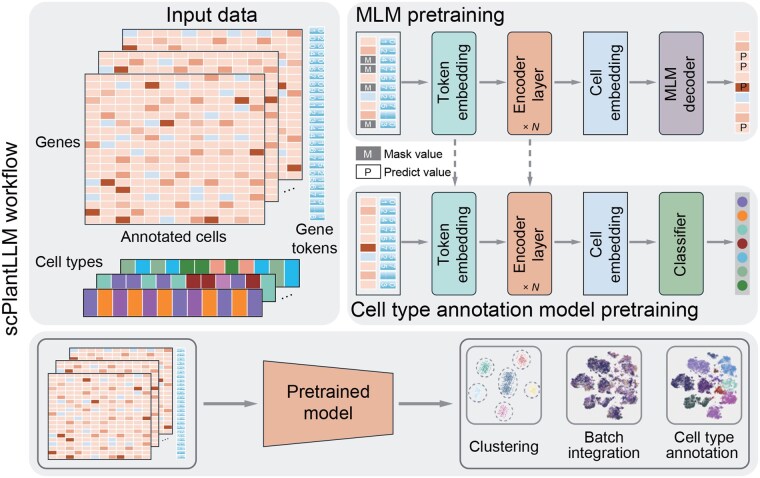

## Introduction

The advent of single-cell RNA sequencing (scRNA-seq) has opened new avenues for understanding the cellular complexity within plant tissues. This technique allows for the dissection of gene expression at the resolution of individual cells, providing unprecedented insights into cellular heterogeneity, developmental processes, and responses to environmental stimuli [1]. Recently, the generation of single-cell data across various plant species has been rapidly increasing. For instance, millions of cells from the model species *Arabidopsis thaliana* have already undergone single-cell transcriptomic profiling [2]. However, the vast volume and complexity of single-cell data present significant analytical challenges, including batch integration, cell type annotation, and gene regulatory network (GRN) inference [3]. Overcoming these challenges necessitates the development of sophisticated bioinformatics tools capable of extracting meaningful biological insights.

The recent advances of foundation models have demonstrated remarkable capabilities in capturing complex patterns within large-scale single-cell datasets, particularly in human studies [4–6]. Inspired by these breakthroughs, we introduce Single-cell Plant Large Language Model (scPlantLLM), a transformer-based model [7] specifically designed for the exploration of single-cell expression atlases in plants. By conceptualizing single cells as “sentences” and genes as “words”, scPlantLLM leverages this analogy to unravel intricate relationships and patterns within plant single-cell data. Tailored to meet the specific needs of plant biologists, scPlantLLM facilitates the efficient exploration and interpretation of large-scale single-cell datasets in key model organisms, such as *Arabidopsis.*

## Method

### Data source

All pretraining datasets in *Arabidopsis* were retrieved from the scPlantDB database (https://biobigdata.nju.edu.cn/scPlantDB/) [2]. The datasets processed by scPlantLLM can be accessed at https://biobigdata.nju.edu.cn/scPlantLLM/. Other publicly available datasets reused in this study were retrieved from the Gene Expression Omnibus (GEO: GSE122687, GSE236290, and GSE157757) and the Genome Sequence Archive (GSA: CRA004082) [8].

### Token embedding

Following the preprocessing steps described in File S1, we independently embedded the gene IDs and their corresponding binned gene expression values. The embedding process employs the nn.Embedding layer in PyTorch [9], where the gene IDs and the binned expression values are independently embedded and subsequently summed to obtain the final token embedding. Let $E_{id}\left(g_{j}\right)$ represent the embedding of the gene ID $g_{j}$ and $E_{\mathrm{expr}}(\text{bin}(x_{ij}))$ represent the embedding of the binned expression value $\text{bin}\left(x_{ij}\right)$. The token embedding for the j-th gene in the i-th cell is then given by:


(1) $${\text{Token}}_{ij}=E_{id}\left(g_{j}\right)+E_{\mathrm{expr}}\left(\text{bin}\left(x_{ij}\right)\right)$$

These token embeddings are subsequently used in model training and inference, providing a robust representation that captures both the identity of the gene and its expression level.

### Encoder layer

The encoder in our model is implemented as a standard six-layer Transformer [7] architecture. Each Transformer layer includes a multi-head self-attention block, with eight attention heads. This multi-head approach enables the model to capture the diverse and complex relationships within the gene expression data by attending to multiple representation subspaces simultaneously.

Since each cell is represented as a “cell sentence” composed of gene IDs and their corresponding binned expression values, the self-attention mechanism is particularly effective in learning intricate dependencies between different genes within each cell. This mechanism is further enhanced by a position-wise fully connected feed-forward network, which applies non-linear transformations to the outputs of the attention layers. This design improves the model’s capacity to learn and generalize complex patterns in the gene expression data, ultimately leading to more accurate predictions and analyses.

### Sequential pretraining strategy

**Figure 1:**
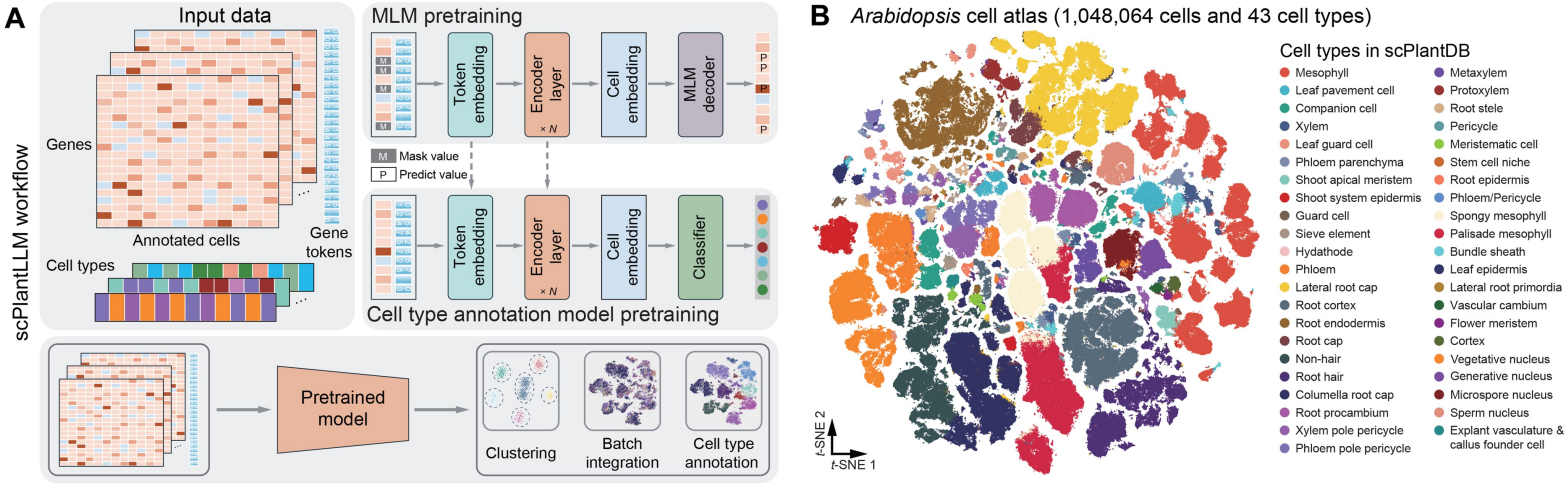
scPlantLLM as a foundation model for exploring plant single-cell expression atlases **A**. The workflow of scPlantLLM. The workflow of scPlantLLM begins with scRNA-seq data, where each cell is transformed into a “cell sentence” composed of gene tokens, with corresponding gene expression values. These inputs are fed into the model, where token embeddings are generated. The data are then processed through an encoder layer, composed of Transformer architecture, to learn cell embeddings. The model is trained using an MLM decoder for predicting masked values and a classifier for pretraining cell type annotations. The final pretrained model can be used for downstream tasks such as clustering, batch integration, and cell type annotation. **B**. The *t*-SNE visualization of the pretrained scPlantLLM cell embeddings, colored by annotated cell types from scPlantDB [2]. The clear separation of cell types emphasizes scPlantLLM’s capacity to learn biologically meaningful patterns from large-scale single-cell data. scPlantLLM, Single-cell Plant Large Language Model; scRNA-seq, single-cell RNA sequencing; MLM, masked language model; *t*-SNE, *t*-distributed stochastic neighbor embedding.

Our training process follows a sequential pretraining strategy, composed of two distinct stages: the masked language model (MLM) pretraining and the cell type annotation pretraining (**Figure 1**A). Each stage is designed to progressively enhance the model’s ability to capture and represent the complex relationships inherent in scRNA-seq data.

#### MLM pretraining

The initial stage involves the application of an MLM [10] approach. In this stage, 15% of the gene expression values within the input sequences are randomly masked, and the model is tasked with predicting these masked values based on the context provided by the remaining unmasked genes. The objective of this stage is to minimize the reconstruction error, which is quantified by the mean squared error (MSE) loss.

#### Cell type annotation pretraining

Following MLM pretraining, the model undergoes a second phase focusing on cell type annotation. The representations learned during the MLM stage are leveraged to classify cells into their respective types. The classification task aims to optimize accuracy by minimizing the cross-entropy loss.

This two-stage pretraining strategy enables the model to develop a robust understanding of gene expression patterns within individual cells and to accurately distinguish between different cell types.

## Results

### Overview of scPlantLLM

The workflow of scPlantLLM, illustrated in Figure 1A, employs a sequential pretraining strategy. Initially, the model is pretrained on large-scale scRNA-seq datasets (Table S1) from our scPlantDB database [2], encompassing over one million *Arabidopsis* cells with manual annotations. This foundational training enables scPlantLLM to capture rich gene expression patterns across diverse cell types. Subsequently, the model undergoes specialized fine-tuning tailored for specific tasks, including scRNA-seq data integration, cell type annotation, and GRN prediction. Upon pretraining, the cell embeddings generated by scPlantLLM can be utilized for clustering, visualization, and cell type labeling. We visualized these embeddings across pretrained datasets using *t*-distributed stochastic neighbor embedding (*t*-SNE) and found that distinct cell types accurately represented by unique clusters, each highlighted in different colors (Figure 1B). This observation underscores the effectiveness of scPlantLLM in capturing and distinguishing the complex cellular diversity within *Arabidopsis* tissues.

### Evaluation of scPlantLLM for cell type annotation

**Figure 2:**
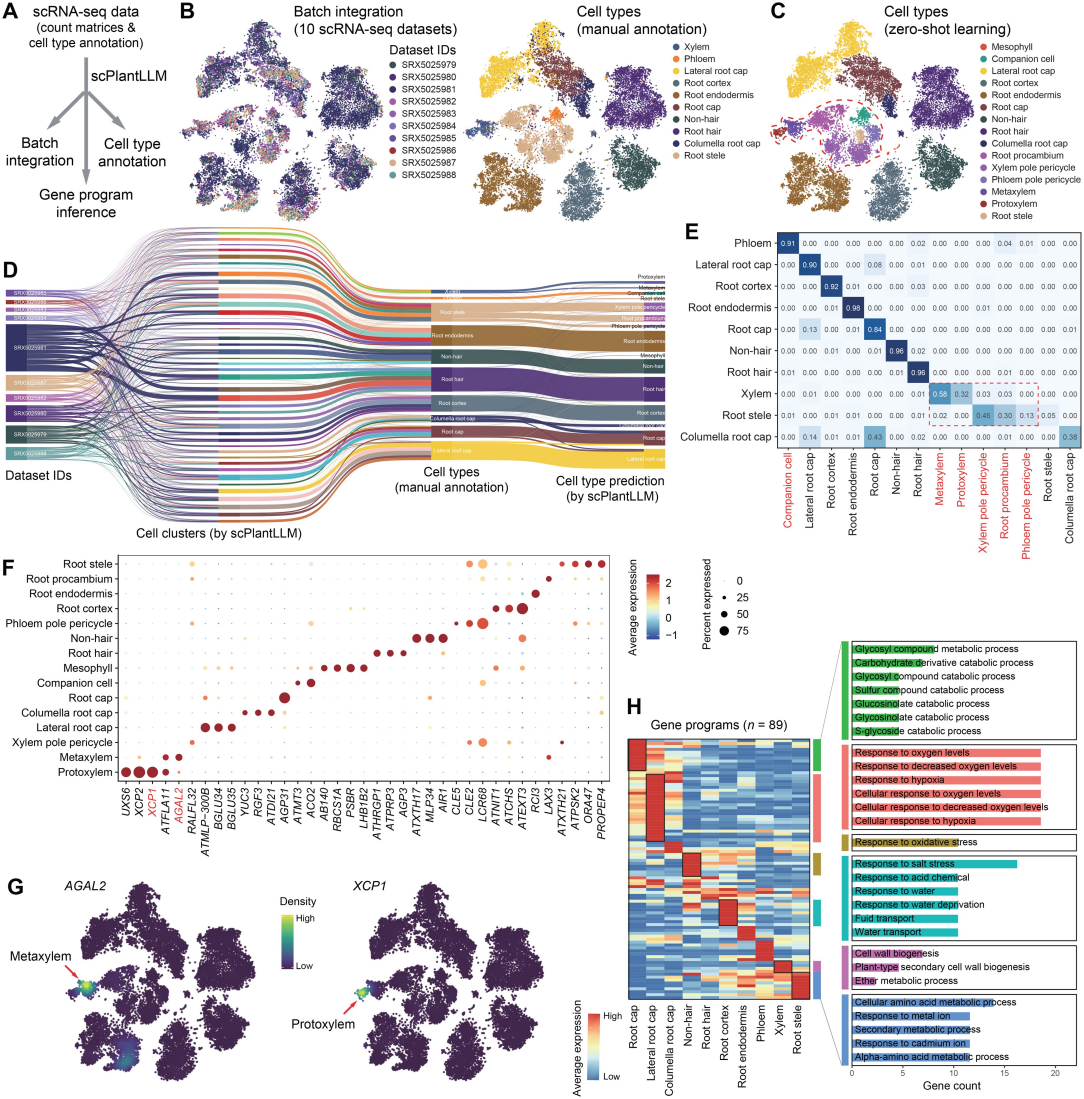
Zero-shot learning analysis of the GSE122687 dataset with scPlantLLM **A**. Workflow for analyzing the scRNA-seq dataset using scPlantLLM. **B**. Data integration and batch correction by scPlantLLM. The *t*-SNE plots show the learned cell embeddings for the GSE122687 dataset, with cells colored by experimental batches (left) and annotated cell types (right). **C**. Cell type prediction by scPlantLLM based on zero-shot learning. The difference of cell type prediction by scPlantLLM from manual annotation in (B) is highlighted in the dashed frame. **D**. Sankey diagram illustrating cell relationships among batches, scPlantLLM-predicted cell clusters, manual annotations, and zero-shot predictions by scPlantLLM. **E**. Confusion matrix showing the agreement between predicted cell types and manual annotations using zero-shot learning. The y-axis represents the manual annotations, while the x-axis represents the predicted cell types. The difference of cell types between manual annotation and prediction is highlighted in red. **F**. Dot plot displaying representative marker genes across cell types. The dot size represents the fraction of cells expressing each gene, while the color intensity indicates the relative expression levels of the genes. Two representative genes highlighted in red are shown in (G). **G**. Feature plots showing the expression of selected marker genes in protoxylem and metaxylem cells. **H**. Cell type-specific gene programs (*n* = 89; left) derived from the learned gene token embeddings and the corresponding enriched biological pathways (right) for selected cell types (color bars).

To evaluate the performance and adaptability of scPlantLLM on new datasets, we selected two independent test datasets, the GSE122687 dataset [11] from root tissue and the GSE236290 dataset [12] from pollen tissue. These datasets were deliberately excluded from the pretraining phase to ensure an unbiased assessment, serving as a benchmark to evaluate the model’s capabilities in key tasks such as data integration, cell type annotation, and gene program inference (**Figure 2**A). We first assessed scPlantLLM’s ability to integrate scRNA-seq data with batch correction, aiming to determine its effectiveness in harmonizing data from different experimental conditions. The integration assessment focused on the model’s ability to achieve consistent and accurate cell cluster or type identification across diverse datasets, ensuring reliable integration and analysis of heterogeneous data. scPlantLLM demonstrated notable integration performance on the independent test datasets without fine-tuning (Figure 2B), underscoring the robustness and generalizability of its pretraining.

**Figure 3:**
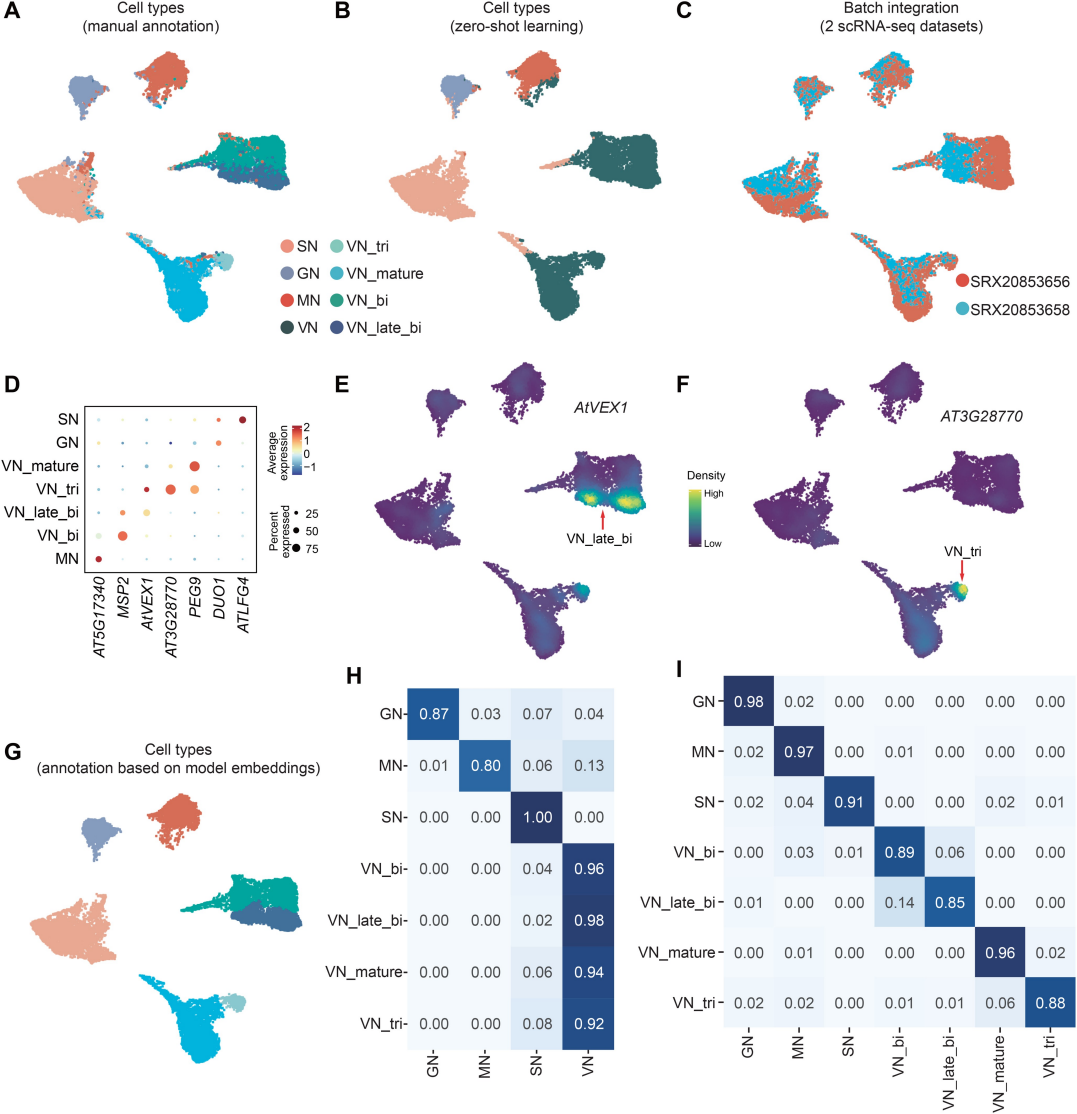
Zero-shot learning analysis of the GSE236290 dataset with scPlantLLM **A**. UMAP visualization showing cell types based on manual annotation. **B**. Cell type prediction by scPlantLLM based on zero-shot learning. **C**. UMAP visualization showing batch integration across two scRNA-seq datasets. **D**. Dot plot displaying representative marker genes across cell types. **E**. UMAP visualization of the expression pattern for the *AtVEX1* gene, highlighting VN_late_bi. **F**. UMAP visualization of the expression pattern for the *AT3G28770* gene, highlighting VN_tri. **G**. UMAP visualization showing cell types annotated based on model embeddings. **H**. Confusion matrix showing the agreement between predicted cell types and manual annotations using zero-shot learning. The y-axis represents the manual annotations, and the x-axis represents the predicted cell types. Since the training set does not include VN subtypes, predictions like VN_bi are classified as VN. **I**. Confusion matrix showing the agreement between the re-annotated cell types using model embeddings from zero-shot predictions. UMAP, uniform manifold approximation and projection; SN, sperm nucleus; GN, generative nucleus; MN, microspore nucleus; VN, vegetative nucleus; VN_tri, vegetative nucleus from tricellular pollen; VN_mature, vegetative nucleus from mature pollen; VN_bi, vegetative nucleus from bicellular pollen; VN_late_bi, vegetative nucleus from late bicellular pollen.

**Figure 4:**
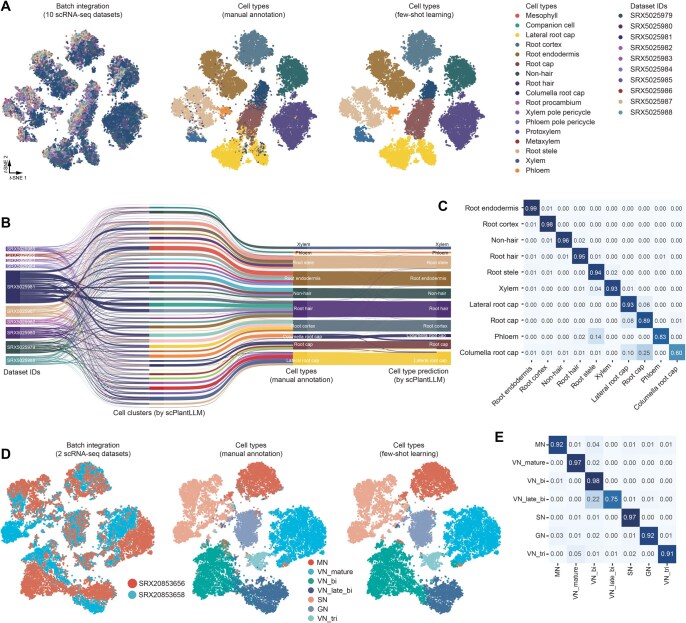
Fine-tuning analysis of datasets using scPlantLLM **A**. *t*-SNE plots illustrating the cell clusters after fine-tuning of scPlantLLM on the GSE122687 dataset, with cells colored by predicted annotations. **B**. Sankey diagram visualizing the flow of cell annotations in the GSE122687 dataset, connecting dataset IDs, cell clusters, manual annotations, and scPlantLLM’s predictions following fine-tuning. **C**. Confusion matrix evaluating the accuracy of cell type predictions by the fine-tuned scPlantLLM model on the GSE122687 dataset, displaying agreements and discrepancies with manual annotations. The y-axis represents the manual annotations, and the x-axis represents the predicted cell types. **D**. *t*-SNE plots illustrating the cell clusters after fine-tuning of scPlantLLM on the GSE236290 dataset. **E**. Confusion matrix evaluating the accuracy of cell type predictions by the fine-tuned scPlantLLM model on the GSE236290 dataset.

Next, we investigated whether the cell embeddings generated by scPlantLLM can be effectively adapted for cell type annotation in new scRNA-seq datasets. Specifically, we assessed whether these embeddings, pretrained on large-scale *Arabidopsis* data, could accurately transfer to and enhance the annotation of cell types in different or previously unseen datasets. To evaluate this, we applied scPlantLLM to the independent test datasets using two distinct strategies: zero-shot learning (Figure 2 and **Figure 3**) and fine-tuning (**Figure 4**). In the zero-shot approach, also known as reference mapping, the pretrained model was used directly to annotate cell types without any additional training. In contrast, the fine-tuning strategy involved further training on a small number of cells from the new dataset to optimize the model’s performance for the specific data. scPlantLLM demonstrated high prediction precision across most cell types on the GSE122687 dataset, with scores of 0.74 for zero-shot learning and 0.93 for fine-tuning setting. Interestingly, in the zero-shot scenarios, scPlantLLM predicted a greater number of cell types compared to the manual annotations (Figure 2B and C), as reflected in the confusion matrix (Figure 2E), with each predicted cell type validated by known marker genes (Figure 2F; Table S2). These additional predictions often corresponded to subtypes or closely related cell types that were also present in the training set and identified in the manual annotations (Figure S1), indicating that scPlantLLM can capture and distinguish subtle cellular subpopulations without additional training. For example, xylem cells were predicted as protoxylem and metaxylem cells — two subtypes of xylem that were already present in the training set, validated by known marker genes such as *XCP1* and *AGAL2* [13–15], respectively (Figure 2G). Adjusting for these nuanced subtype annotations, the prediction accuracy could reach up to 0.91 in the zero-shot setting. This suggests that, while fine-tuning aims to improve model performance, it does not always enhance accuracy and may sometimes introduce errors. On the other hand, due to the limitations of the training dataset, certain cell subtypes were not represented. However, the model’s predictions were still able to group these subtypes into a distinct cluster. For example, in the zero-shot learning predictions on the GSE236290 dataset, the model grouped subtypes such as vegetative nucleus from bicellular pollen (VN_bi), vegetative nucleus from late bicellular pollen (VN_late_bi), and vegetative nucleus from tricellular pollen (VN_tri) under a common label, vegetative nucleus (VN) (Figure 3B and H). To further validate the accuracy of these predictions, we performed clustering and findMarker analyses on the embeddings generated by the model. The results indicated that these cell subtypes indeed exhibited clear clustering features (Figure 3D–F). Based on this, we re-annotated the dataset by combining the model’s embeddings with known marker genes, ultimately finding that the model’s prediction accuracy was approximately 0.92 (Figure 3G and I). To further evaluate the model’s applicability across different species, we employed transfer learning by transferring the model pretrained on *Arabidopsis* to the maize and rice datasets. After transfer learning, zero-shot predictions were evaluated using two independent datasets. The results indicated that the maize model achieved a prediction accuracy of 0.97, while the rice model achieved a prediction accuracy of 0.87 (Figure S2). These findings demonstrate that, after transfer learning, the model can still effectively annotate cell types across different species. The aforementioned findings highlight scPlantLLM’s capability to generalize across datasets, accurately capturing intricate cellular hierarchies and enhancing annotation precision in new scRNA-seq data.

### Evaluation of scPlantLLM for batch integration

**Figure 5:**
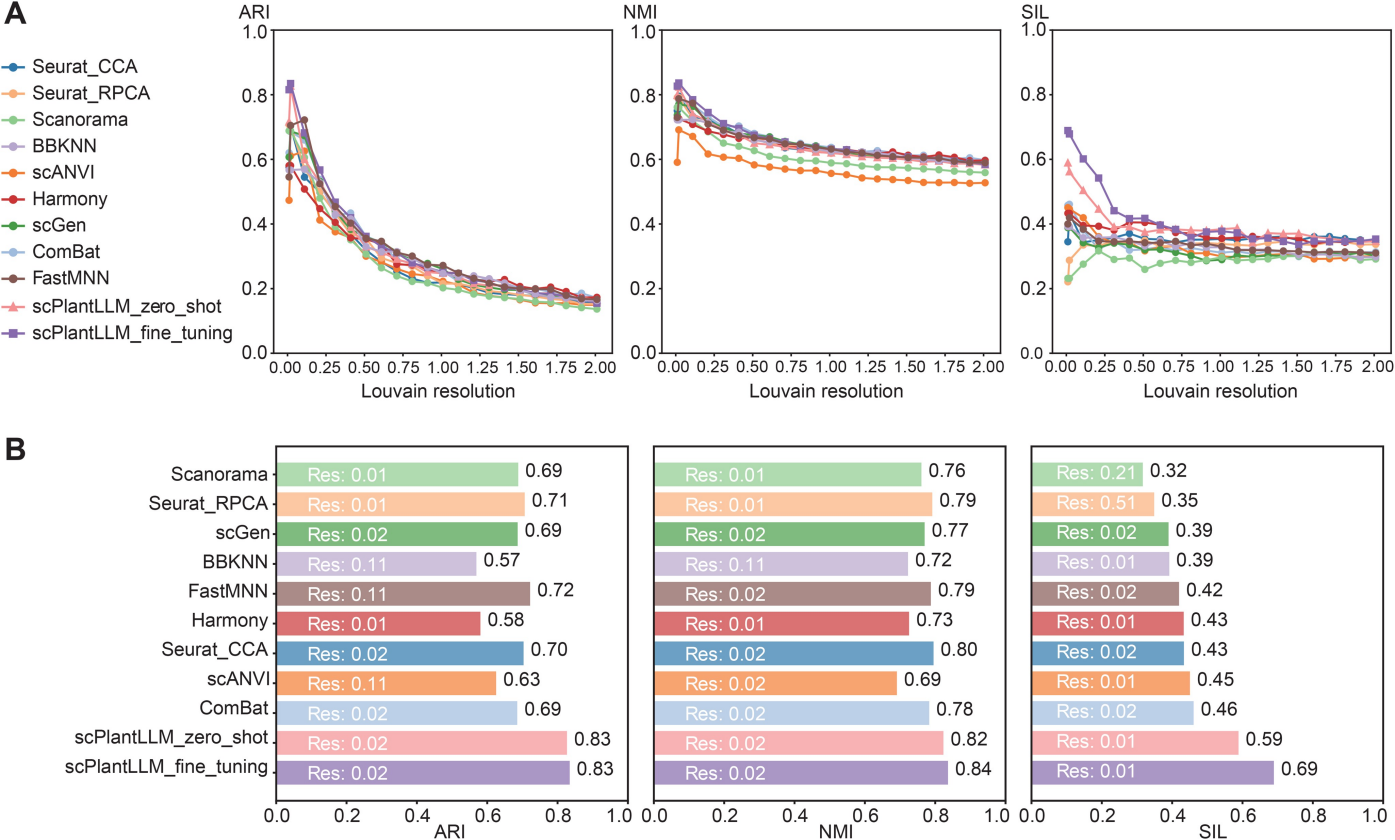
Performance evaluation of scPlantLLM compared to other batch integration methods on the GSE122687 dataset **A**. Line plots illustrating the performance of each method across varying Louvain clustering resolution values. **B**. Bar plots showing the optimal scores achieved by each method for ARI, NMI, and SIL metrics. ARI, adjusted rand index; NMI, normalized mutual information; SIL, silhouette score; Res, resolution.

Moreover, in both zero-shot and fine-tuning settings, scPlantLLM consistently outperformed other batch integration methods on the GSE122687 dataset, achieving the highest scores across key metrics including adjusted rand index (ARI) [16], normalized mutual information (NMI) [17], and silhouette score (SIL) [18], which highlights its superior clustering accuracy and ability to effectively distinguish cell types compared to alternative approaches (**Figure 5**A and B). Specifically, scPlantLLM’s superior ARI scores reflect its precise clustering capability, while its top performance in NMI and SIL highlights its effectiveness in preserving biologically meaningful patterns and maintaining clear boundaries between cell types.

### Gene embeddings and attention maps reveal gene–gene interactions

Furthermore, beyond generating cell embeddings, scPlantLLM also encodes feature relationships through gene embeddings and attention maps (File S1), leveraging these mechanisms to capture intricate gene–gene interactions and regulatory dynamics in plant cells. Specifically, the attention map constructs a gene network that represents and highlights gene–gene interactions within the dataset, unveiling the underlying functional relationships and regulatory connections among genes. Additionally, gene embeddings capture distinctive gene program activation patterns across various cell states, providing insights into how gene interactions and regulatory mechanisms change in response to different cellular conditions. Therefore, these features enable a more comprehensive understanding of gene programs, and their expression dynamics derived from scPlantLLM. In the GSE122687 dataset, scPlantLLM can successfully extract various gene programs with cell type-specific activation patterns using gene embeddings (Figure 2H). These cell type-specific gene programs are enriched in biological pathways associated with specific cellular functions. For instance, gene programs for the root cap are involved in sensing and responding to various oxygen-related conditions, including hypoxia and decreased oxygen levels.

**Figure 6:**
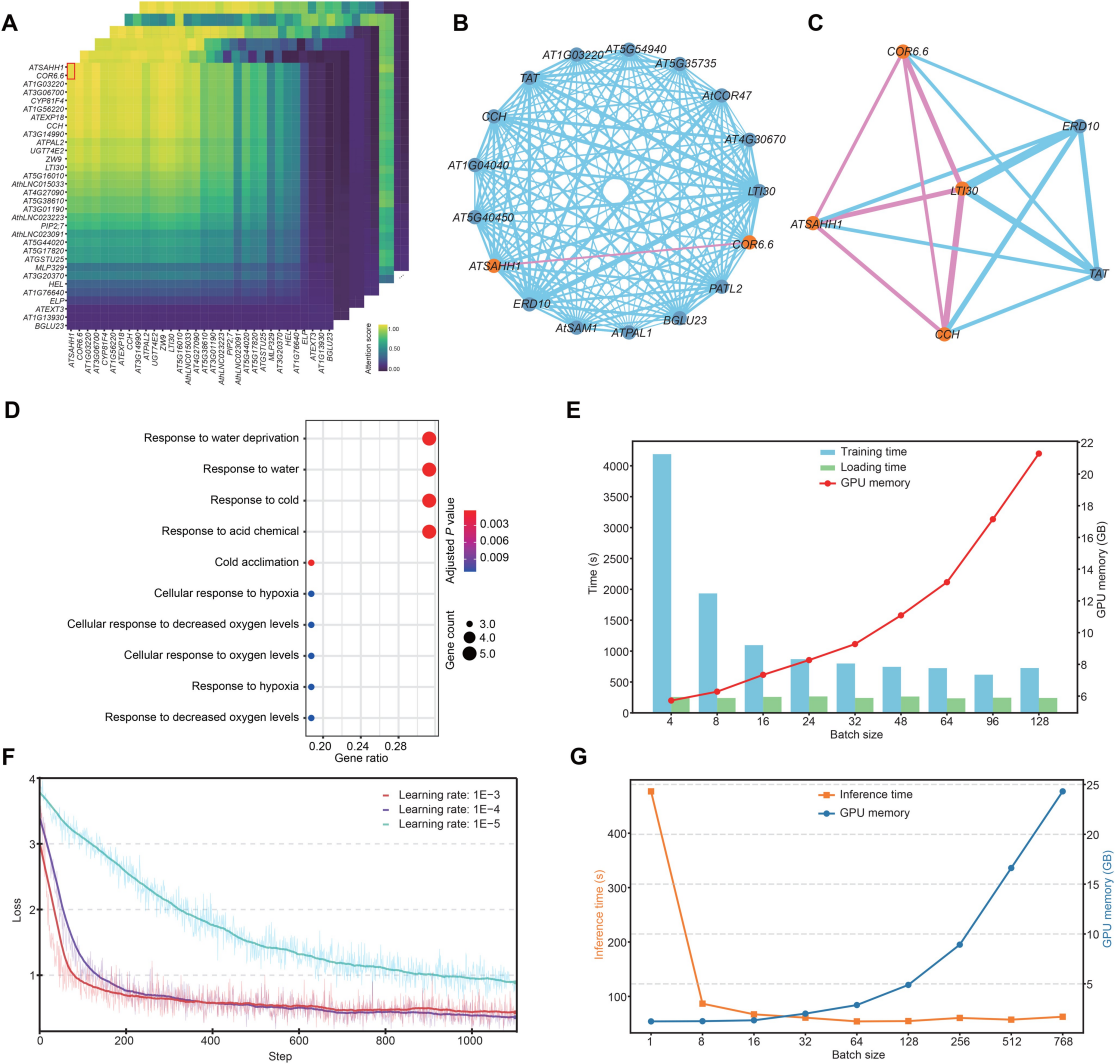
Analysis and visualization of gene regulatory networks as well as the model performance **A**. Heatmap displaying normalized attention scores (0–1) between gene pairs, with color intensity representing the scores: yellow for higher attention and purple for lower scores. **B**. Gene regulatory network derived from the first cell sample, centered on *COR6.6* and *ATSAHH1*, showing their interactions with the top 5 related genes and illustrating multi-level relationships with a depth of 2. **C**. Subgraph of (B), focusing on the top 2 related genes and limiting the depth to 1 to highlight direct interactions. **D**. Dot plot showing the top enriched biological processes from Gene Ontology analysis, with each dot representing a biological process. The analysis includes genes from the gene regulatory network in (B). **E**. Bar plot showing the relationship between batch size and various performance metrics during training. **F**. scPlantLLM training loss profiles under different learning rates. **G**. Plot showing the inference time and GPU memory usage with varying batch sizes.

To further explore gene–gene interactions in an individual cellular context, we randomly selected a single-cell sample in the GSE122687 dataset to construct a gene regulatory network (**Figure 6**). Through the analysis of normalized attention scores within the cell (Figure 6A), *COR6.6* and *ATSAHH1* were identified as central regulatory hub genes. A multi-layered interaction network was constructed, linking these hub genes to their five most strongly related genes (Figure 6B). This network revealed clusters of co-regulated genes and highlighted the potential role of these hubs in driving critical biological processes within the cell. To further refine the analysis, a subgraph (Figure 6C) was generated by reducing the GRN to include the two most strongly related genes for each central hub and limiting the network depth to one. This subgraph emphasized the most significant direct regulatory relationships, with genes such as *ATSAHH1*, *LTI30*, *COR6.6*, and *CCH* exhibiting strong correlations. The literature indicates that *ATSAHH1* is a key gene associated with methylation, and plays a crucial role in plant growth, development, and stress response [19]. *LTI30* and *COR6.6* are two cold-induced genes that enhance plant cold tolerance by stabilizing cellular structures, such as membranes, and contributing to osmotic adjustment, making them essential components of the cold stress response [20]. *CCH* encodes a copper chaperone protein that facilitates copper transport, aiding plants in combating oxidative stress and maintaining redox homeostasis [21]. Enrichment analysis results show significant enrichment of biological processes related to cold stress, dehydration response, and oxygen fluctuation within the gene network, indicating that these genes may work together to regulate key biological processes in plant stress responses (Figure 6D). The strong correlations among these genes suggest that they may collaborate to form functional networks that collectively regulate plant cell differentiation and stress responses. To further validate the model, we conducted an additional experiment using the GSE236290 dataset, randomly selecting a single-cell sample (VN, vegetative nucleus of pollen) and constructed a gene regulatory network based on the cell’s attention score (Figure S3). GO analysis revealed that the network was significantly enriched in biological processes related to DNA conformation change. DNA conformation change plays an important role in the development of pollen cells and generative cells, potentially related to chromosomal rearrangement and cell cycle regulation [22]. This suggests that the selected genes may play a key regulatory role in plant growth, development, and stress responses. By constructing GRNs and uncovering critical gene interactions, the model has proven its capability to resolve complex regulatory relationships at the single-cell level, providing a powerful tool for understanding the dynamic regulatory mechanisms underlying plant cellular functions and stress adaptations.

### Impact of hyperparameter choices on model performance

To assess the impact of hyperparameter choices on model performance, we investigated the effects of different batch sizes and learning rates on training time, GPU memory usage, and inference time. Increasing the batch size during training led to higher GPU memory consumption. Notably, while larger batch sizes resulted in shorter training time, the data loading time remained relatively unchanged. To balance training efficiency and GPU memory usage, we selected a batch size of 64 (Figure 6E). To evaluate the impact of different learning rates on loss, we used a training subset of 500,000 cells and observed the changes with a batch size of 64 over one epoch (Figure 6F). The results showed that the lower learning rate (1E−4) achieved more stable convergence with a lower final loss. In contrast, the higher learning rate (1E−3) resulted in faster convergence but a higher final loss, while the learning rate of 1E−5 exhibited slower convergence. Based on these results, we selected a learning rate of 1E−4 as the final choice to balance training speed and model performance. During inference predictions, increasing the batch size led to a significant rise in GPU memory consumption. For smaller batch sizes, GPU memory usage remained relatively stable; however, when the batch size exceeded 64, GPU memory increased sharply. Meanwhile, inference time stabilized substantially once the batch size surpassed 64 (Figure 6G). These results highlight the trade-off between batch size, inference efficiency, and GPU memory usage.

## Discussion

This study introduces scPlantLLM, a robust artificial intelligence model for plant scRNA-seq analysis. With its sequential pretraining strategy, scPlantLLM demonstrates exceptional adaptability, accurately identifying cell types, integrating datasets, and uncovering regulatory networks. The choice of masked language models (MLM) [10] as the pretraining architecture stems from their ability to effectively learn contextual relationships and generalize across diverse scRNA-seq datasets, making them particularly well-suited for tasks such as zero-shot cell type annotation. However, MLMs are less effective for modeling relational structures, such as cell-cell interactions or gene regulatory networks (GRNs). In contrast, graph neural networks (GNNs) excel in capturing such relationships, making them valuable for analyzing spatial transcriptomics and GRNs [23], while variational autoencoders (VAEs) are particularly suited for tasks like dimensionality reduction and batch correction capabilities [24]. Although MLMs are highly effective in sequence-based tasks, integrating GNNs and VAEs into scPlantLLM could enhance its capability to analyze spatial and multi-modal data, while retaining the robust contextual learning power of MLMs. The hybrid framework combining these models holds promise for advancing scRNA-seq analysis by providing a more comprehensive approach to cellular and regulatory complexity.

scPlantLLM achieved an impressive zero-shot accuracy of 0.90 on independent datasets, effectively capturing nuanced subtypes like protoxylem and metaxylem, underscoring its ability to annotate new datasets and resolve cellular hierarchies in complex plant systems. Transfer learning demonstrated the model's adaptability across species, showcasing its versatility in cell type annotation for maize and rice. Despite its strong performance across species, the relatively smaller training samples for maize and rice compared to *Arabidopsis* may still require additional fine-tuning or dataset expansion to further improve cross-species performance. As seen in Figure S2, while scPlantLLM excels in cell type annotation, batch integration did not meet expectations, which may be a focus for future optimization. Compared to alternative integration methods, scPlantLLM consistently outperformed in clustering accuracy and biological relevance, as shown by its superior ARI, NMI, and SIL scores. Additionally, our hyperparameter evaluation revealed trade-offs between batch size, inference efficiency, and GPU memory usage. A batch size of 64 strikes a balance between training efficiency and memory usage, while a learning rate of 1e-4 ensures stable convergence and lower final loss. These insights will guide future model optimization in resource-constrained environments. In conclusion, scPlantLLM represents a powerful tool for cell type annotation, offering valuable insights into plant cell biology. Future research will focus on further enhancing its accuracy and cross-species applicability.

## Code availability

The source code for scPlantLLM is available at GitHub (https://github.com/compbioNJU/scPlantLLM). The code has also been submitted to BioCode at the National Genomics Data Center (NGDC), China National Center for Bioinformation (CNCB) (BioCode: BT007822), which is publicly accessible at https://ngdc.cncb.ac.cn/biocode/tools/BT007822.

## CRediT author statement

**Guangshuo Cao:** Methodology, Software, Formal analysis, Writing – original draft. **Haoyu Chao:** Formal analysis. **Wenqi Zheng:** Investigation, Validation. **Yangming Lan:** Investigation, Validation. **Kaiyan Lu:** Investigation. **Yueyi Wang:** Investigation. **Ming Chen:** Writing – review & editing. **He Zhang:** Writing – review & editing. **Dijun Chen:** Conceptualization, Supervision, Writing – review & editing. All the authors have read and approved the final manuscript.

## Competing interests

The authors declare no competing interests.

## Supplementary Material

qzaf024_Supplementary_Data
