# Supplementary material for: scPlantLLM: A Foundation Model for Exploring Single-cell Expression Atlases in Plants
Source: Genomics Proteomics Bioinformatics. 2025 Mar 17;23(3):qzaf024. doi: 10.1093/gpbjnl/qzaf024 (PMC12417071; doi:10.1093/gpbjnl/qzaf024)
Supplement: qzaf024_Supplementary_Data [file qzaf024_supplementary_data.zip › File S1.docx]

**File S1 Supplementary method**

**Method**

**Training set and test set**

We used a collection of datasets comprising one million single-cell RNA-seq samples of Arabidopsis thaliana from scPlantDB [1]. For model training, 90% of these cells were used, with the remaining 10% reserved for validation. To rigorously assess the model’s performance, we randomly selected two external datasets, GSE122687 dataset [2] and GSE236290 dataset [3], as external test sets.

For cross-species transfer learning, the maize and rice data were also obtained from scPlantDB, with the rice dataset containing 427,077 cells and the maize dataset containing 449,530 cells. 90% of the data were used for training and 10% for validation. To evaluate model performance, we randomly selected two independent experimental sets from the GSE157757 dataset [4] and CRA004082 dataset [5]datasets as validation sets. To ensure the reliability of the predictions, we filtered out cell types with fewer than 35 cells during the model prediction process.

**Data preprocessing**

The scPlantDB provides annotations for 54 cell types in Arabidopsis thaliana single-cell data from multiple tissues (Table S3). However, some annotations were related to the cell cycle, such as "Unknown," "S phase," "G2/M phase," "G1/G0 phase," and "G1/S phase." These cell types were filtered out, leaving us with 43 distinct cell types.

To effectively represent single-cell RNA sequencing (scRNA-seq) data, we first extracted the raw count matrix from each SRX project. These SRX projects are associated with their corresponding SRP accession numbers, which represent broader research initiatives (BioProjects). The raw data obtained from these SRX projects served as the foundation for our analysis (Table S1).

Next, we constructed a dictionary that maps all gene names to their corresponding gene IDs. For each scRNA-seq matrix, we selected 3,000 highly variable genes (HVGs) and used the dictionary to map these gene names to their respective gene IDs. In this framework, each cell was treated as a "cell sentence," where the 3,000 HVGs represented the "words" within that sentence.

In this study, we opted for binning to discretize gene expression values instead of applying normalization techniques like log- or scale-normalization. This approach aimed to reduce noise while preserving key biological patterns, enhancing interpretability and enabling downstream models to capture essential variations across cells. Discretized values effectively reflected the distribution characteristics of gene expression levels, as detailed in the following steps:

Let $X\in\mathbb{R}^{N\times G}$ represent the gene expression matrix, where $N$ denotes the number of cells and $G$ denotes the number of genes. For any given gene expression value $x_{ij}$ (the expression value of the $j$th gene in the $i$th cell), we first calculate the minimum non-zero expression value $\text{min}_{+}$ and the maximum expression value $\text{max}$:

| $\text{min}_{+}=min\left( \left\{ x_{ij} \right\vert x_{ij}>0\} \right), \text{max}=max\left( X \right)$ | $\left( 1 \right)$ |
| --- | --- |

Next, we divide the range of expression values into $B$ discrete intervals, with bin edges defined as:

| $\text{bin\_edges}=\{b_{k}{\}}_{k=0}^{B} \text{where} b_{k}=\text{min}_{+}+k\cdot\frac{\text{max}-\text{min}}{B-1}$ | $\left( 2 \right)$ |
| --- | --- |

$k$ is the index of the bin and ranges from $0$ to $B$, where $B$ represents the total number of bins. Each $b_{k}$ represents the boundary of the $k$-th bin used for discretizing the gene expression values.

For each expression value $x_{ij}$, if $x_{ij}>0$, it is mapped to the nearest interval, and the value is replaced by the corresponding bin index:

| $x_{ij}\to\text{bin}\left( x_{ij} \right)=k \text{where} b_{k}\leq x_{ij}<b_{k+1}$ | $\left( 3 \right)$ |
| --- | --- |

The mapping process can be formally expressed as:

| $\text{binned\_value}\left( x_{ij} \right)=\left\{ \begin{matrix} 0 & \text{if }x_{ij}=0 \\ \text{bin}\left( x_{ij} \right) & \text{otherwise} \end{matrix} \right.$ | $\left( 4 \right)$ |
| --- | --- |

After discretization, genes with an expression value of zero, along with their corresponding gene IDs, were removed from the dataset to retain only those with meaningful expression levels for further analysis.

To ensure consistency in data input, the length of each cell’s sequence of gene IDs and their corresponding binned expression values was standardized to a fixed length of 1,500. If a sequence was shorter than this length, it was padded with a special token <pad>. For sequences exceeding this length, we randomly sampled three subsets of the sequence at proportions of 0.25, 0.5, and 0.75 of the original length. Each sampled subset was treated as an independent cell and included in the training set.

After preprocessing, the dataset was split into training and validation sets, and we ultimately retained 1,048,064 cells for training.

**Loss function of sequential pretraining strategy**

Mean Squared Error (MSE) loss function is defined as:

| $L_{MSE}=\frac{1}{N}\sum_{i=1}^{N} \left( y_{i}-\hat{y}_{i} \right)^{2}$ | $\left( 6 \right)$ |
| --- | --- |

where $y_{i}$ represents the true gene expression value, $\hat{y}_{i}$ is the predicted value, and $N$ is the number of masked values.

Cross-Entropy loss is expressed as:

| $L_{CE}=-\frac{1}{N}\sum_{i=1}^{N} \sum_{c=1}^{C} y_{i,c}\log\left( \hat{y}_{i,c} \right)$ | $\left( 7 \right)$ |
| --- | --- |

where $y_{i,c}$ is a binary indicator $\left( 0\text{ or }1 \right)$ signifying whether cell type $c$ is correct for instance $i$, $\hat{y}_{i,c}$ is the predicted probability of instance $i$ belonging to cell type $c$, and $C$ is the total number of cell types.

**Decoder Layer**

During the masked language model pretraining phase, the decoder is implemented as a multi-layer perceptron (MLP). Similarly, in the cell type annotation model pretraining phase, the classifier is also realized using an MLP. Both the decoder and classifier share a common architecture: each consists of several fully connected layers, where the input passes through successive linear transformations interleaved with activation functions. These layers gradually transform the encoded representation into the final output format, whether it be predicting the masked values in the language model pretraining phase or classifying the cell types during the annotation phase.

**Batch integration**

After pretraining, the cell embeddings generated by the model can be directly utilized for batch integration. The model integrates data from different experimental batches into a unified representational space, achieving tight alignment of the same cell types across batches while maintaining distinct separation between different cell types. This approach effectively mitigates batch effects—non-biological variations introduced by differences in sample processing, sequencing techniques, or other technical factors [6]. In practical applications, the cell embeddings produced during the pretraining stage already sufficiently capture the characteristics of different cell types, making them a solid foundation for batch integration. As a result, cells of the same type from different batches naturally cluster together, while cells of different types remain appropriately distinct. This method not only simplifies the batch integration process but also enhances the model’s efficiency and accuracy in handling cross-batch data.

To evaluate the performance of scPlantLLM in batch integration, we compared it to several widely used batch correction methods, including Seurat V3 (CCA, RPCA) [7], scANVI [8], Scanorama [9], ScGen [10], Harmony [11], Combat [12], and BBKNN [13], which represent state-of-the-art approaches in single-cell data integration. All methods were applied to the same dataset for a fair comparison, and their performance was assessed using three widely recognized metrics: Adjusted Rand Index (ARI) [14], Normalized Mutual Information (NMI) [15], and Silhouette Coefficient (SIL) [16]. These metrics evaluate the quality of integration by measuring the alignment of similar cell types across batches and the separation of distinct cell types.

**Cell type annotation**

Following batch integration, the pretrained cell embeddings are directly used for subsequent cell type annotation tasks. A classifier leverages these embeddings to categorize cells, aiming to accurately identify their types. The classifier can be fine-tuned or retrained in the integrated embedding space to ensure accurate recognition of different cell types within the unified representational space.

To further refine the prediction results, we perform Leiden clustering [17] on the model-generated embeddings. This step groups cells into distinct clusters based on the learned embeddings. For each cluster, the most frequent predicted cell type is assigned as the final cell type for all cells within that cluster. This approach ensures that the final cell type annotation reflects both the model’s predictions and the cluster-specific structure of the data, thus improving the robustness and accuracy of cell type assignments.

**Gene regulatory network analysis via attention maps**

*Extraction of attention maps*

For each cell, we extracted the attention matrices from the final layer of the Transformer model. The attention matrix dimensions correspond to the number of genes in the input, with each element representing the attention weight between a pair of genes. The attention weight indicates the importance of one gene to another within the model, which can be used to infer potential regulatory relationships between genes. A higher attention weight generally implies that the model perceives these genes to have a stronger interaction or regulatory relationship in a specific biological context. To reduce noise and emphasize potentially important gene interactions, we selected only the top-k genes with the highest attention scores. In this study, we chose the top 32 genes, determined based on predefined thresholds or the highest percentage of attention scores.

*Frequency aggregation of gene pairs*

To construct a comprehensive view of the gene regulatory network, we aggregated the attention scores across all cells. We used a frequency-based aggregation strategy: when a gene pair exhibited an attention score exceeding a specific threshold in a sufficient number of cells, it was considered significant. In this study, we defined significance as gene pairs that appeared in at least 20 cells with a normalized attention score greater than 0.5. This approach allowed us to capture stable gene interactions across different biological conditions. Specifically, we maintained a sparse frequency matrix where each non-zero entry represented a gene pair that met the attention threshold in numerous cells, along with the frequency of occurrence.

*Construction of the gene regulatory network (GRN)*

The aggregated significant gene pairs were then used to construct the gene regulatory network (GRN). We utilized the Python library NetworkX for graph analysis, creating a graph where nodes represent individual genes and edges represent significant regulatory relationships between gene pairs. The edge weights corresponded to the normalized frequency of significant attention scores across all cells, reflecting the strength and consistency of each interaction. To visualize the GRN, we extracted subgraphs centered on key genes and visualized the interactions within a specified depth. We adjusted the edge thickness according to the connection weights to visually represent the strength of the gene-gene interactions.

**Transfer learning**

Transfer learning is a machine learning method that involves applying a model, which has already been trained on one task, to another related task in order to improve learning efficiency and performance on the new task. In the context of plant single-cell data modeling, transfer learning is especially valuable because, while gene names differ across species, the underlying gene functions and expression patterns may be highly similar. For example, in gene expression data, although different species may use different identifiers for the same gene, their biological functions and expression patterns may be conserved.

In our scPlantLLM model, due to the differences in gene names between species, the model cannot directly perform zero-shot predictions across species. Therefore, transfer learning provides a solution by transferring the pre-trained model from Arabidopsis to other plant species, significantly reducing the training requirements for new species data.

*Steps in transfer learning*

Load pre-trained parameters from Arabidopsis model
Arabidopsis thaliana is an important model organism with a rich dataset from genomic studies. We first train a base model on the Arabidopsis dataset. The gene expression patterns learned by this model serve as the foundation for transfer learning. During transfer learning, we load the parameters that have already been trained on the Arabidopsis model, particularly the encoder part.

Modify the decoder
Since each plant species has a unique set of genes, we need to adjust the structure of the decoder to accommodate the gene set of the target species. Specifically, the Multi-Layer Perceptron (MLP) layer in the decoder needs to be modified according to the number of cell types in the target species (i.e., the number of classes of cells in the target species). This means that during transfer learning, we will adjust the output dimension of the decoder based on the number of cell types in the target species.

Keep other hyperparameters unchanged
Aside from modifying the decoder structure, other hyperparameters of the model, such as learning rate, batch size, etc., will remain unchanged to ensure that we retain as much of the pre-trained knowledge from the Arabidopsis model as possible. This helps avoid instability or performance degradation caused by excessive modifications in other parts of the model.

Fine-tuning the model
Once the parameter adjustments are made, the model will be fine-tuned using the data from the target species. During fine-tuning, the model will optimize the parameters of the decoder based on the single-cell data from the target species, while keeping the encoder part of the model fixed or making only minimal adjustments. This approach leverages the prior knowledge from the Arabidopsis model, reducing the need for large amounts of data from the target species and thus improving training efficiency.

**Implementation details**

The model consists of six Transformer blocks [18], each with eight self-attention heads, producing an embedding dimension of 512. The Decoder includes three linear layers by default: the first two map inputs to the embedding dimension (512), followed by ReLU activation and layer normalization for stability and faster convergence. The final layer maps embeddings to the target class space. For sequential pretraining, the model was trained with a batch size of 64 and a learning rate of 0.0001 using the Adam optimizer. The Masked Language Modeling phase lasted 10 epochs, while the Cell Type Annotation phase ran for 20, with the best validation performance observed at epoch 9 of the latter phase. For fine-tuning, the same training configuration was adopted, including the optimizer (Adam), learning rate (0.0001), batch size (64), and learning rate scheduling. For cross-species transfer learning, the same parameters were used, with each species trained for 5 epochs.

The model was implemented using PyTorch [19], and all analyses were performed in a Python 3.11 environment. Since the scPlantDB data is stored in RDS format, the Seurat package in R was used to extract counts matrices and HGV data from SRX files. Data preprocessing was primarily conducted in Python with numpy, pandas, and scikit-learn. For Gene Regulatory Network inference, NetworkX was used for graph construction and visualization, while Seaborn and Matplotlib generated attention score heatmaps and GRN visualizations. The adjustText library optimized label placement to reduce clutter in network graphs. All experiments were performed on a platform with four NVIDIA 4090 GPUs, 1 TB RAM, and an Intel Xeon Silver 4310 CPU.

In batch integration, three metrics were computed across a range of Louvain clustering [20] resolution values to assess the stability and robustness of each method at different levels of clustering granularity. This allowed for a comprehensive evaluation of each method’s ability to align similar cell types while keeping different cell types distinct. Specifically, we utilized the scanpy [21] along with scikit-learn [15] to implement Louvain clustering and compute the evaluation metrics. Louvain clustering was performed using the scanpy function sc.tl.louvain, which allowed us to vary the resolution parameter to adjust the granularity of the clustering.

**References**

[1] He Z, Luo Y, Zhou X, Zhu T, Lan Y, Chen D. scPlantDB: a comprehensive database for exploring cell types and markers of plant cell atlases. Nucleic Acids Res 2024;52:D1629–38.

[2] Shulse CN, Cole BJ, Ciobanu D, Lin J, Yoshinaga Y, Gouran M, et al. High-throughput single-cell transcriptome profiling of plant cell types. Cell Rep 2019;27:2241–7.

[3] Wang S, Wang M, Ichino L, Boone BA, Zhong Z, Papareddy RK, et al. MBD2 couples DNA methylation to transposable element silencing during male gametogenesis. Nat Plants 2024;10:13–24.

[4] Bezrutczyk M, Zoellner NR, Kruse CPS, Hartwig T, Lautwein T, Koehrer K, et al. Evidence for phloem loading via the abaxial bundle sheath cells in maize leaves. Plant Cell 2021;33:531–47.

[5] Wang Y, Huan Q, Li K, Qian W. Single-cell transcriptome atlas of the leaf and root of rice seedlings. J Genet Genomics 2021;48:881–98.

[6] Tran HTN, Ang KS, Chevrier M, Zhang X, Lee NYS, Goh M, et al. A benchmark of batch-effect correction methods for single-cell RNA sequencing data. Genome Biol 2020;21:12.

[7] Stuart T, Butler A, Hoffman P, Hafemeister C, Papalexi E, Mauck WM, III, et al. Comprehensive integration of single-cell data. Cell 2019;177:1888–902.

[8] Xu C, Lopez R, Mehlman E, Regier J, Jordan MI, Yosef N. Probabilistic harmonization and annotation of single-cell transcriptomics data with deep generative models. Mol Syst Biol 2021;17:e9620.

[9] Hie B, Bryson B, Berger B. Efficient integration of heterogeneous single-cell transcriptomes using Scanorama. Nat Biotechnol 2019;37:685–91.

[10] Lotfollahi M, Wolf FA, Theis FJ. scGen predicts single-cell perturbation responses. Nat Methods 2019;16:715–21.

[11] Korsunsky I, Millard N, Fan J, Slowikowski K, Zhang F, Wei K, et al. Fast, sensitive and accurate integration of single-cell data with Harmony. Nat Methods 2019;16:1289–96.

[12] Johnson WE, Li C, Rabinovic A. Adjusting batch effects in microarray expression data using empirical Bayes methods. Biostatistics 2007;8:118–27.

[13] Polanski K, Young MD, Miao Z, Meyer KB, Teichmann SA, Park JE. BBKNN: fast batch alignment of single cell transcriptomes. Bioinformatics 2020;36:964–5.

[14] Hubert L, Arabie P. Comparing partitions. J Classifi 1985;2:193–218.

[15] Pedregosa F, Varoquaux G, Gramfort A, Michel V, Thirion B, Grisel O, et al. Scikit-learn: machine learning in python. J Mach Learn Res 2011;12:2825–30.

[16] Rousseeuw PJ. Silhouettes - a graphical aid to the interpretation and validation of cluster-analysis. J Comput Appl Math 1987;20:53–65.

[17] Traag VA, Waltman L, van Eck NJ. From Louvain to Leiden: guaranteeing well-connected communities. Sci Rep 2019;9:5233.

[18] Vaswani A, Shazeer N, Parmar N, Uszkoreit J, Jones L, Gomez AN, et al. Attention is all you need. 31^st^ annual conference on neural information processing systems (NIPS) 2017.

[19] Paszke A, Gross S, Massa F, Lerer A, Bradbury J, Chanan G, et al. PyTorch: an imperative style, high-performance deep learning library. 33^rd^ conference on neural information processing systems (NeurIPS) 2019.

[20] Blondel VD, Guillaume J-L, Lambiotte R, Lefebvre E. Fast unfolding of communities in large networks. Journal of Statistical Mechanics-Theory and Experiment 2008:P10008.

[21] Wolf FA, Angerer P, Theis FJ. SCANPY: large-scale single-cell gene expression data analysis. Genome Biol 2018;19:15.
