# Supplementary material for: scPlantLLM: A Foundation Model for Exploring Single-cell Expression Atlases in Plants
Source: Genomics Proteomics Bioinformatics. 2025 Mar 17;23(3):qzaf024. doi: 10.1093/gpbjnl/qzaf024 (PMC12417071; doi:10.1093/gpbjnl/qzaf024)
Supplement: qzaf024_Supplementary_Data [file qzaf024_supplementary_data.zip › supplementary material captions.docx]

**Supplementary material**

**File S1 Supplementary method**

**Figure S1 Visualization of selected marker genes in *Arabidopsis thaliana* cell types**

The t-SNE plots display the expression patterns of marker genes, with high expression regions indicated by increased density (yellow-green scale). Red arrows highlight specific cell types.

**Figure S2 Cross-species zero-shot learning analysis using scPlantLLM**

**A.** UMAP visualization of scPlantLLM’s zero-shot learning predictions on CRA004082 dataset after transfer learning on *rice* (*Oryza sativa*): The left panel shows manual annotations, the middle panel shows zero-shot predictions, and the right panel shows batch integration across two scRNA-seq datasets. **B.** UMAP visualization of scPlantLLM’s zero-shot learning predictions on GSE157757 dataset after transfer learning on *rice* (*Zea mays*). **C.** Confusion matrix showing the agreement between predicted cell types and manual annotations using zero-shot learning on CRA004082 dataset. The y-axis represents the manual annotations, and the x-axis represents the predicted cell types. **D.** Confusion matrix showing the agreement between predicted cell types and manual annotations using zero-shot learning on GSE157757 dataset.

**Figure S3 Construction and visualization of gene regulatory networks based on attention maps on GSE236290 dataset**

**A.** Heatmap displaying normalized attention scores (0–1) between gene pairs. **B.** Gene regulatory network derived from the first cell sample, centered on *GYRB3* and *ATCIMS*, showing their interactions with the top 5 related genes and illustrating multi-level relationships with a depth of 2. **C.** Subgraph of (B), focusing on the top 2 related genes and limiting the depth to 1 to highlight direct interactions. **D.** Dot plot showing the top enriched biological processes from Gene Ontology (GO) analysis, with each dot representing a biological process. The analysis includes genes from the gene regulatory network in panel B.

**Table S1 Comprehensive data summary for scPlantLLM model training**

**Table S2 Known marker genes from the *Arabidopsis thaliana* scRNA-seq dataset**

**Table S3 Overview of cell counts across organs and tissues from scPlantDB**
