# Supplementary figures and images for: scPlantLLM: A Foundation Model for Exploring Single-cell Expression Atlases in Plants
Source: Genomics Proteomics Bioinformatics. 2025 Mar 17;23(3):qzaf024. doi: 10.1093/gpbjnl/qzaf024 (PMC12417071; doi:10.1093/gpbjnl/qzaf024)

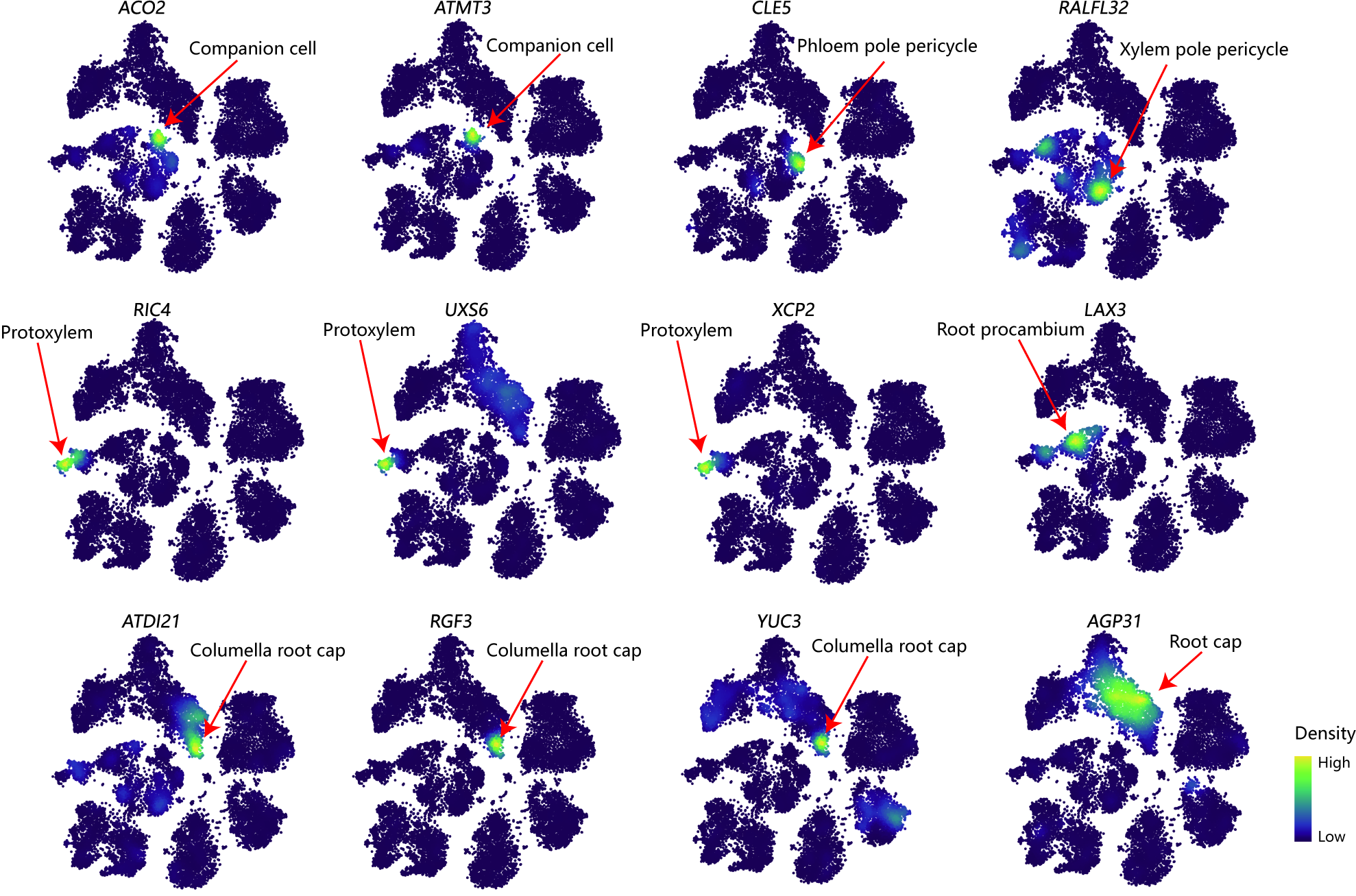

Supplement: qzaf024_Supplementary_Data [file qzaf024_supplementary_data.zip › Figure S1.tif]

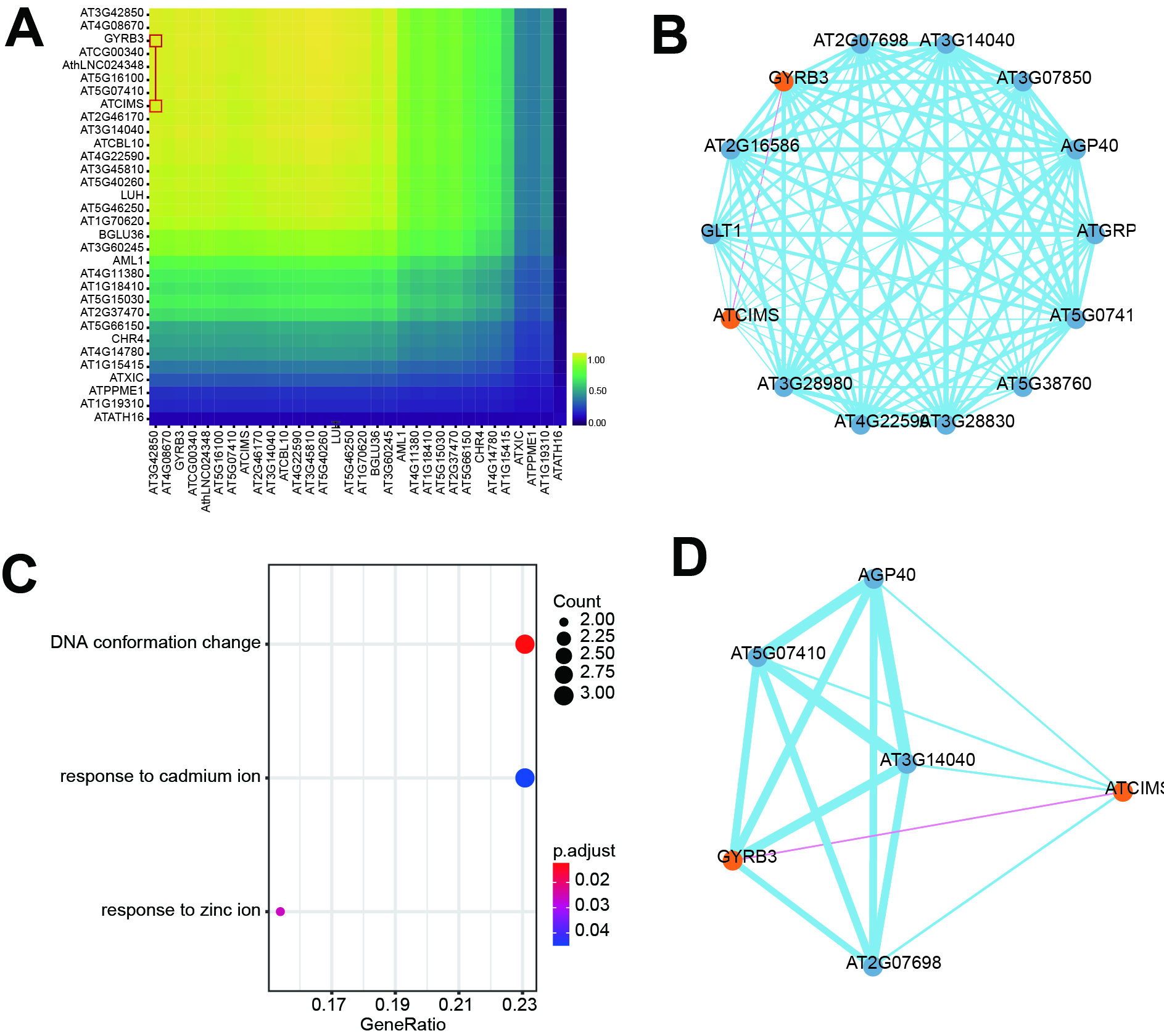

Supplement: qzaf024_Supplementary_Data [file qzaf024_supplementary_data.zip › Figure S2.jpg]

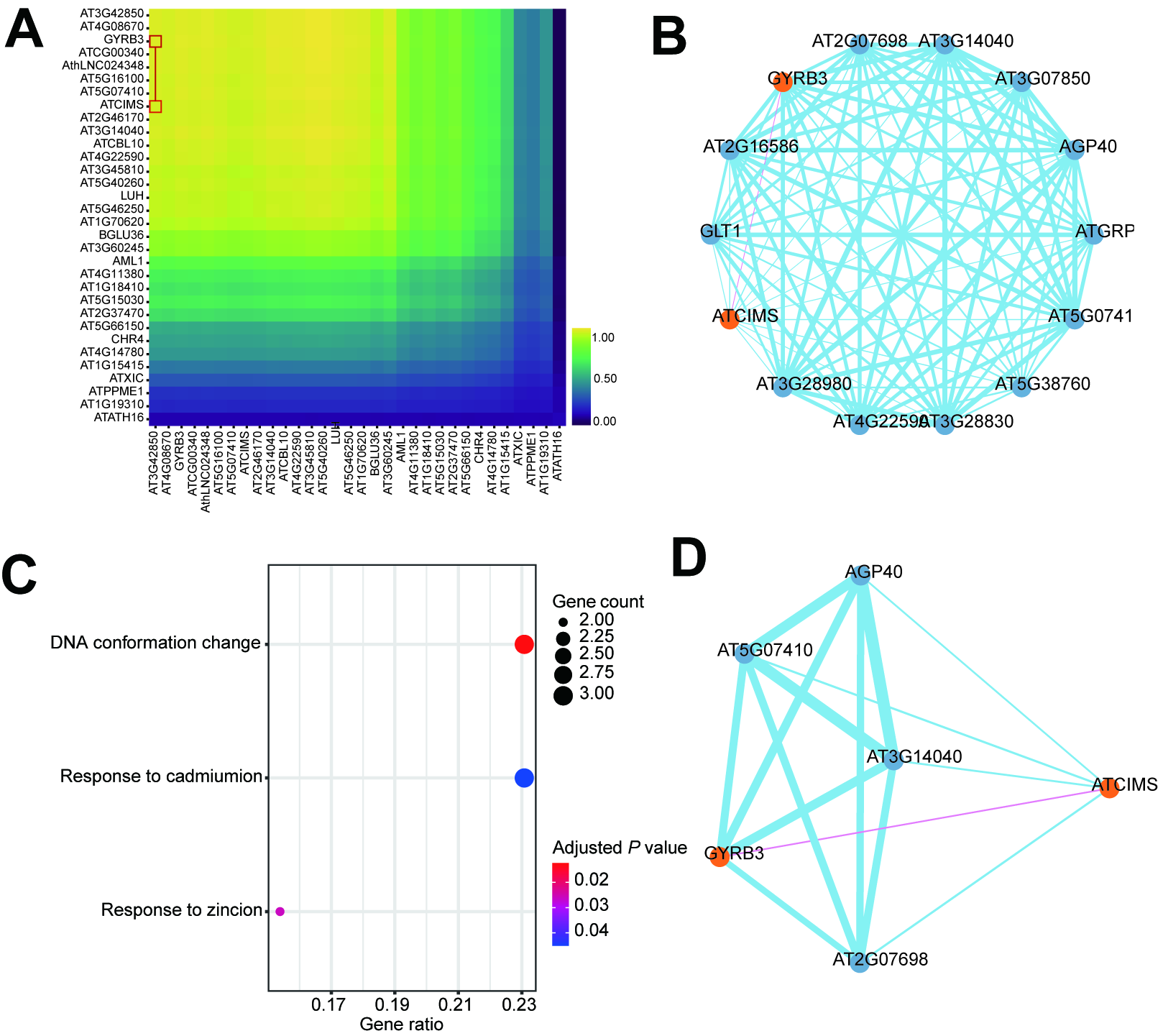

Supplement: qzaf024_Supplementary_Data [file qzaf024_supplementary_data.zip › Figure S3.tif]
